# Predicting lincRNA-Disease Association in Heterogeneous Networks Using Co-regularized Non-negative Matrix Factorization

**DOI:** 10.3389/fgene.2020.622234

**Published:** 2021-01-12

**Authors:** Yong Lin, Xiaoke Ma

**Affiliations:** ^1^School of Physics and Electronic Information Engineering, Ningxia Normal University, Guyuan, China; ^2^School of Computer Science and Technology, Xidian University, Xi'an, China

**Keywords:** disease-lincRNA association, non-negative matrix factorization, heterogeneous network, regularization, network analysis

## Abstract

Long intergenic non-coding ribonucleic acids (lincRNAs) are critical regulators for many complex diseases, and identification of disease-lincRNA association is both costly and time-consuming. Therefore, it is necessary to design computational approaches to predict the disease-lincRNA associations that shed light on the mechanisms of diseases. In this study, we develop a co-regularized non-negative matrix factorization (aka *Cr-NMF*) to identify potential disease-lincRNA associations by integrating the gene expression of lincRNAs, genetic interaction network for mRNA genes, gene-lincRNA associations, and disease-gene associations. The Cr-NMF algorithm factorizes the disease-lincRNA associations, while the other associations/interactions are integrated using regularization. Furthermore, the regularization does not only preserve the topological structure of the lincRNA co-expression network, but also maintains the links “lincRNA → gene → disease.” Experimental results demonstrate that the proposed algorithm outperforms state-of-the-art methods in terms of accuracy on predicting the disease-lincRNA associations. The model and algorithm provide an effective way to explore disease-lncRNA associations.

## 1. Introduction

Long intergenic non-coding RNAs (lincRNAs) are transcripts whose lengths are greater than 200 nucleotides with little or no protein coding potential (Kapranov et al., 2007; Mercer et al., 2009; Wang and Chang, 2011). In the traditional view, lncRNAs are considered as “junk RNAs” because they do not code protein sequences. However, it has been proven that many lncRNAs are dysregulated in human cancers and implicated in disease progression through modulating apoptosis, increasing cellular oncogenic potential, or inhibiting tumor growth (Wilusz et al., 2009; Taftet al., 2010).

With the advent of the next generation sequencing (NGS) techniques, a large number of lincRNAs have been identified (Guttman et al., 2009, 2010; Wang et al., 2009; Popadin et al., 2013), providing a great opportunity to investigate the functions of lncRNAs. Unfortunately, very few lincRNAs have been depicted with explicit molecular mechanisms in cancers through biological experiments or computational approaches (Guo et al., 2013; Zhao et al., 2016; Tang et al., 2017). Thus, discovering lincRNA patterns that are associated with cancers is urgently needed as it sheds light on the underlying mechanism of diseases.

Therefore, great efforts have been devoted to investigating the functions or patterns of lincRNAs by analyzing omics data, such as DNA sequences, expression profiles, and genomic annotations. For instance, Liao et al. (2011) constructed a co-expression network for protein-coding genes and lincRNAs, and predicted the functions of lincRNAs via analyzing the constructed co-expression network. However, it has been criticized because of the fact that the gene expression profile cannot fully characterize the connections between genes and lincRNAs. To overcome this problem, Guo et al. (2013) developed a global prediction algorithm to infer probable functions of lincRNAs at a large scale by integrating gene expression, a protein-protein interaction (PPI) network, and DNA sequences. Ma et al. (2017a) designed a pipeline to discover disease related lincRNA modules across various clinical stages of cancers, rather than predicting the functions of lincRNAs. Ning et al. (2016) extracted the disease associated with SNPs within human lincRNAs.

Despite numerous research contributions to extract various patterns of lincRNAs, few efforts have been devoted to analyzing lincRNA-disease associations, which can be used to predict implicated diseases. The available methods to predict lincRNA-disease associations can be categorized into two classes: biological experiments-based methods and computational based approaches. The biological experiment-based methods have been criticized because they are time-consuming and costly. Computational based approaches are thus an alternative which can provide critical clues for biologists in revealing the mechanisms of diseases.

However, it is non-trivial to design effective and efficient algorithms to predict the lincRNA-disease associations largely due to two reasons. First, to infer the lincRNA-disease associations, large-scale known association data is a prerequisite. Second, diseases, such as cancers, are complex and difficult to characterize. Thus, it is wise to predict the lincRNA-disease associations by integrating omics data with an immediate purpose to improve the accuracy of prediction. Regarding the first concern, as more experimentally validated lincRNA-disease associations accumulate, researchers have summarized these associations as lincRNA-disease database, such as LncRNADisease (Chen et al., 2012) and Lnc2Meth (Zhi et al., 2018). These known associations provide a great opportunity to infer the lincRNA-disease associations.

Regarding the second concern, many algorithms have been developed to address this issue. For example, Yang et al. (2014) predicted the lncRNA-disease associations by constructing two biological networks, such as lncRNA-implicated disease network and disease network. Then, a propagation algorithm is applied to extract similar lncRNAs and diseases from those constructed networks. To integrate the expression profile, Chen et al. (2012) designed the Laplacian regularized least squares for lncRNA-disease associations, where the tissue expression profiles of intergenic lncRNA (lincRNA) from the Human BodyMap LincRNA project (Cabili et al., 2011). Zhang et al. (2017) proposed a label propagation algorithm to predict lncRNA-disease associations by integrating multiple heterogeneous networks. Fu et al. (2018) developed a matrix factorization-based model to predict disease-lncRNA associations, where multiple data matrices from various heterogeneous sources are factorized into low-rank matrices. Lan et al. (2017) designed a web server for the prediction of the lncRNA-disease. These algorithms achieve promising performance in inferring lncRNA-disease associations.

However, all of these studies solely focus on ranking lncRNA-disease associations via integrating the additional features of lncRNA genes and diseases, which cannot make use of the known prior knowledge to further improve the performance of algorithms. The latent features facilitate the identification of biological patterns, such as copy number and driver genes (Xi et al., 2020a,b). Actually, compared to the lincRNAs, knowledge of protein-coding genes is more redundant. How do you effectively incorporate the prior information into algorithms in order to perform a particular function and/or to infer a disease in the biological systems? For instance, Liao et al. (2011) made use of the gene-lncRNA relation to predict the functions of lncRNAs, implying that integration of omic data is promising for improving the performance of algorithms. Recently, Biswas et al. (2015) designed the *iNMF* algorithm by integrating expression profiles of protein-coding and lncRNA genes, lncRNA-disease and gene-disease associations, and gene genetic interaction networks to predict the diseases of lncRNAs. The experimental results demonstrate that it is wise to integrate omics data to infer lncRNA-disease associations a major motivation for this study.

**Figure 1:**
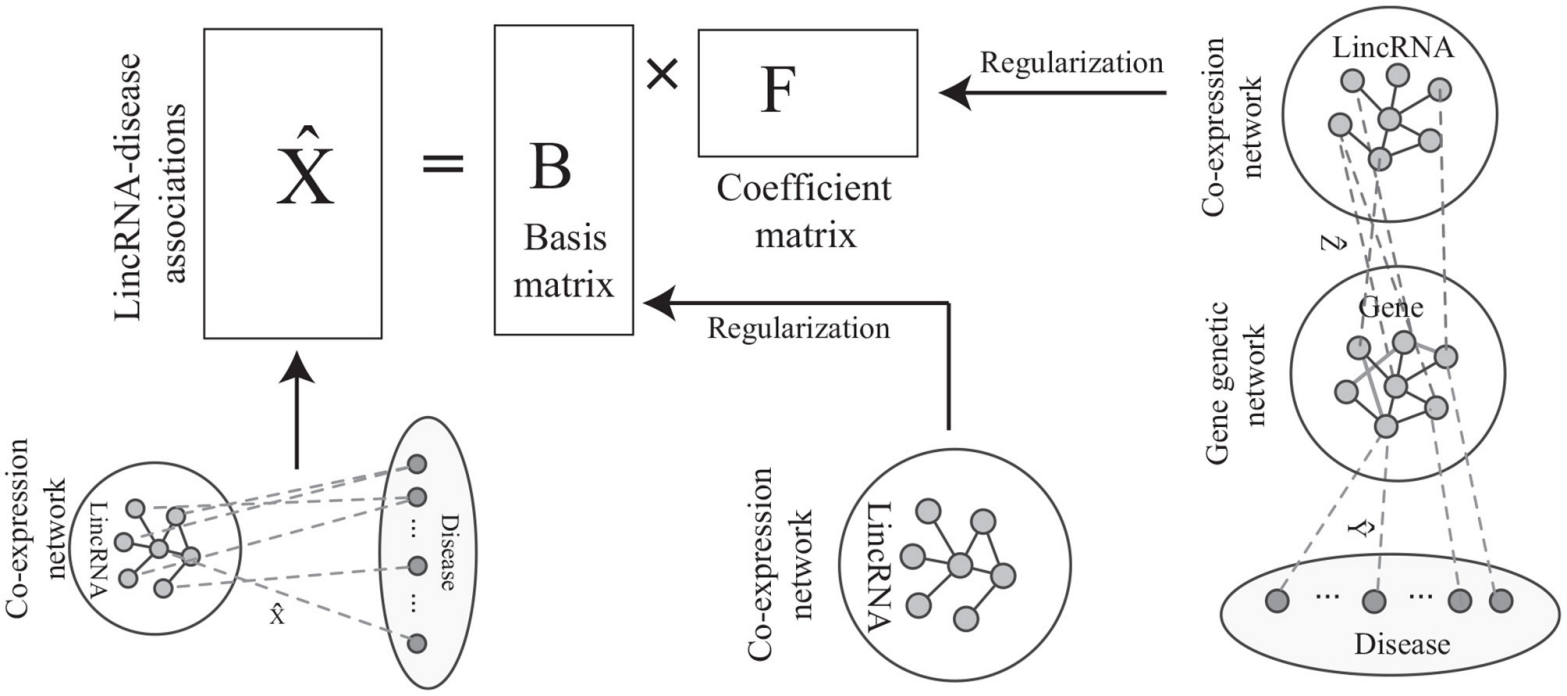
Overview of the Cr-NMF algorithm. It factorizes the known disease-lincRNA associations, regularizing the other associations/interactions. Specifically, the basis matrix is the latent representation of lincRNAs, which preserves the topological structure of lincRNA co-expression networks. The coefficient matrix regularizes the links “lincRNA → gene → disease”.

*iNMF* jointly factorizes expression profiles of lncRNA and protein-coding genes. However, the method ignores the fact that lncRNAs execute their functions via interactions between them. Thus, we develop a novel algorithm, named co-regularized NMF (Cr-NMF), to predict lincRNA-disease associations via the heterogeneous network with multiple types of association, including lincRNA co-expression, lincRNA-disease, gene-disease, gene genetic and lincRNA-gene associations (As shown in Figure 1). The Cr-NMF algorithm decomposes the lincRNA-disease associations into the feature and coefficient matrices; the latent features for lincRNAs regularize the topological structure of lincRNA co-expression network. Furthermore, we also expect that the factorization reflects paths from *lincRNA* → *gene* → *disease*, which is also represented by regularization. Compared to state-of-the-art algorithms, the proposed algorithm is more accurate in the lincRNA-disease prediction. The proposed model and method provide an effective strategy to predict lncRNA-disease associations.

The rest of this study is organized as follows. Section 2 presents the details of the proposed algorithm. Then, in section 3, we set up experiments to validate the performance of Cr-NMF. Finally, conclusions are drawn in section 4.

## 2. Algorithm

The algorithm consists of two major components: the objective function construction and optimization rules, as shown in Figure 1. The procedure and analysis of the proposed algorithm are addressed in this section.

### 2.1. Notations

Before presenting the detailed description of the proposed algorithm, let us introduce some terminologies that are widely used in the sections that follow.

The notations for the algorithm are summarized in Table 1. Let *n*_*g*_ be the number of genes, *n*_*d*_ be the number of diseases, *n*_*l*_ be the number of lincRNAs. The lincRNA co-expression network is denoted by *G*^[*l*]^ = (*V*^[*l*]^, *E*^[*l*]^), where *V*^[*l*]^ is the set of lincRNAs and *E*^[*l*]^ is the interaction sets based on lincRNA co-expression coefficients. The adjacency matrix for *G*^[*l*]^ is denoted by matrix *W*^[*l*]^, where $w_{ij}^{\left[l\right]}$ is the weight on edge (*i, j*) in *G*^[*g*]^. Because *G*^[*l*]^ is undirected, *W*^[*l*]^ is symmetric. The degree of the *i*-th lincRNA in *G*^[*l*]^ is defined as the sum of weights on the edges connecting to it, i.e., $d_{i}=\underset{i}{\sum }w_{ij}^{\left[l\right]}$. The degree matrix of *G*^[*l*]^ is the diagonal one with degree sequence, i.e., $D^{\left[l\right]}=diag\left(d_{i}^{\left[l\right]},\ldots ,d_{n_{l}}^{\left[l\right]}\right)$. Given network *G*^[*l*]^, we construct a normalized Laplacian matrix *L*^[*l*]^ = *I* − (*D*^[*l*]^)^−1/2^*W*^[*l*]^(*D*^[*l*]^)^−1/2^. Analogously, we construct the normalized Laplacian matrix for *G*^[*g*]^ as *L*^[*g*]^ = *I* − (*D*^[*g*]^)^−1/2^*W*^[*g*]^(*D*^[*g*]^)^−1/2^.

**Table 1:** Notations.

**Notation**	**Definition and description**
*n*_*g*_, *n*_*d*_, *n*_*l*_	Number of genes, diseases, and lincRNAs
*G*^[*g*]^	Gene genetic interaction network
*G*^[*l*]^	lincRNA co-expression network
$\hat{X}$	known lincRNA-disease associations
$\hat{Y}$	known gene-disease associations
$\hat{Z}$	genes-lincRNAs associations
*W*^[*g*]^, *W*^[*l*]^	weighted adjacency matrix for *G*^[*g*]^ and *G*^[*l*]^
$w_{ij}^{\left[g\right]}$	the element at *i*-th row *j*th column in matrix *W*^[*g*]^
*D*	the degree diagonal matrix, i.e., *D* = *diag*(*d*_1_, …, *d*_*n*_)
${\bar{W}}^{\left[g\right]}$	normalized *G*^[*g*]^, i.e., ${\bar{W}}^{\left[g\right]}=D^{-1/2}W^{\left[d\right]}D^{-1/2}$
*W*′	transpose of matrix *W*
*w*_*i*._	the *i*-th row of matrix *W*
*w*_.*j*_	the *j*-th column of matrix *W*
||*W*||_*F*_	Frobenius norm of matrix *W*
*Tr*(*W*)	the Tr of matrix *W*, i.e., $Tr\left(W\right)=\underset{i}{\sum }w_{ii}$

The known lincRNA-disease associations are represented by $\hat{X}$, where the row represents a lincRNA and column denotes a disease. The known gene-disease associations are denoted by $\hat{Y}$, where rows correspond to genes and columns denote diseases. Thegene-lincRNA associations $\hat{Z}$ are constructed based expression data, where the rows correspond to genes, columns to lincRNAs, and *z*_*ij*_ = 1 if the *i*-th gene and *j*-th lincRNA are associated with at least one disease, 0 otherwise.

### 2.2. Objective Function

NMF aims at learning the representation parts of the original data (Lee and Seung, 1999) by approximating the target matrix into the product of two low-ranking matrices. Specifically, given matrix *W*, NMF decomposes *W* into two non-negative matrices *B*_(*m*+*n*) × *k*_ and *F*_(*m*+*n*) × *k*_ such that

(1) $$\begin{array}{r} W\approx BF^{\prime },s.t.B\geq 0,F\geq 0, \end{array}$$

where *B* is the basis matrix and *F* is the feature matrix. NMF has been widely applied for graph analysis (Ma et al., 2018a), link prediction (Ma et al., 2017b, 2018b), bioinformatics (Chen and Zhang, 2016; Ma et al., 2016, 2018c).

**Figure 2:**
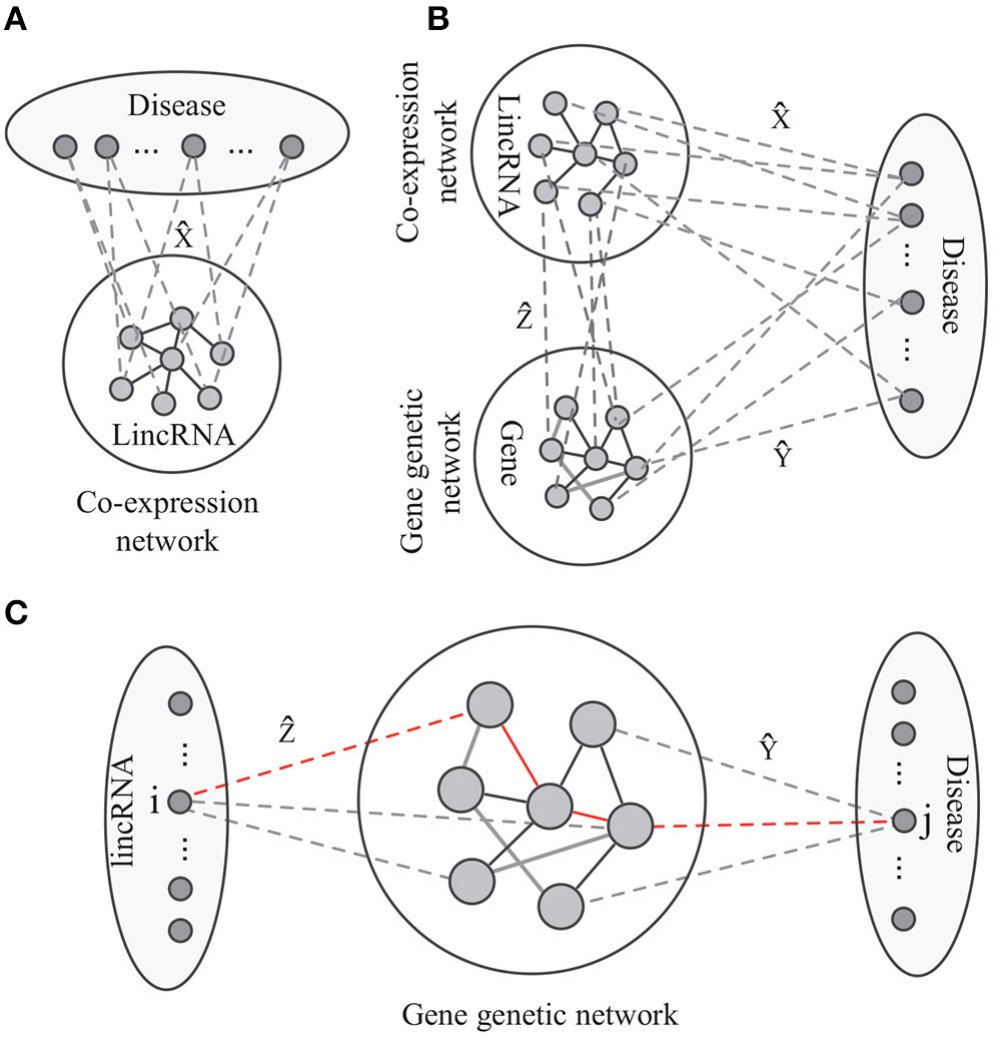
The paradiagrams for disease-lincRNA association prediction. **(A,B)** The label propagation model predicts the disease-lincRNA associations by each single disease based on the lincRNA co-expression network, and **(C)** the proposed algorithm integrates lincRNA co-expression network, gene genetic networks and various associations. The dashed lines show disease-lincRNA association (matrix $\hat{X}$), disease-gene association (matrix $\hat{Y}$), and lincRNA-gene associations (matrix $\hat{Z}$).

As shown in Figure 2A, the label propagation-based model has been widely studied and successfully applied to predict phenotype-gene associations (Hwang and Kuang, 2010; Vanunu et al., 2010; Hwang et al., 2011). The model aims at identifying the disease-lincRNA associations *X* under some constraints. Thus, the objective function of label propagation model is defined as

(2) $$\begin{array}{r} O_{lp}=\theta Tr\left(X^{\prime }L^{\left[l\right]}X\right)+\left(1-\theta \right)||X-\hat{X}|{|}_{F}^{2}, \end{array}$$

where θ ∈ (0, 1) is a parameter to balance the contributions of the two terms, *Tr*(·) is the Tr function and ||·||_*F*_ is the Frobenius norm. To further improve the performance of label propagation model, Petegrosso et al. (2017) proposed transfer learning-based label propagation model to integrate omics data to predict phenome-genome association.

Given the disease-lincRNA associations $\hat{X}$, Cr-NMF first factorizes $\hat{X}$ into the product of matrix *B* and *F*, i.e.,

(3) $$\begin{array}{r} \hat{X}=BF,\;s.t.\;B\geq 0,F\geq 0, \end{array}$$

where $B\in R^{n_{l}\times r}$ is the basis matrix, $F\in R^{r\times n_{d}}$ is the feature matrix, *r* is the number of latent variables (usually, *r* ≪ min{*n*_*l*_, *n*_*d*_}). By casting Equation (3) as an optimization form, we obtain the following objective function as

(4) $$\begin{array}{r} O_{NMF}=\frac{1}{2}||\hat{X}-BF|{|}_{F}^{2},\;s.t.\;B\geq 0,F\geq 0. \end{array}$$

On the one hand, matrix *B* is considered to be the representations of lincRNAs in the latent space, where each row *b*_*i*._ is interpreted as latent representation of the *i*-th lincRNA. We expect the latent representations in matrix *B* preserve the local topological structure of lincRNAs *G*^[*l*]^. Specifically, if a pair of lincRNAs are close in terms of the latent representation, they are well connected in *G*^[*l*]^ and vice versa. Cai et al. (2010) demonstrated that

(5) $$\begin{array}{l} O_{G^{\left[l\right]}}=\frac{1}{2}\underset{i}{\sum }\underset{j}{\sum }||b_{i.}-b_{j.}|{|}^{2}w_{ij}^{\left[l\right]}\\ \;=Tr\left(B^{\prime }D^{\left[l\right]}B\right)-Tr\left(B^{\prime }W^{\left[l\right]}B\right)\\ \;=Tr\left(B^{\prime }L^{\left[l\right]}B\right). \end{array}$$

On the other hand, the disease-lincRNA associations are also related to the topological structure of the gene interaction network, lincRNA-gene association (Figure 2B), and the disease-gene associations. The association between the *i*-th lincRNA and the *j*-th disease follows the pattern *lincRNA* → *gene* → *gene network* → *disease*. For example, in Figure 2C, the *i*-th lincRNA and *j*-th disease are connected by the red path. There is a good biological interpretation for this pattern: the lincRNAs transduce signal to the target genes. The dysfunctional signal possibly leading to an abnormal response via interaction among genes, resulting in diseases. Thus, the disease-lincRNA association *w*_*ij*_ can be defined as a product of weights on all the paths connecting the *i*-th lincRNA and *j*-th disease, i.e.,

(6) $$\begin{array}{r} x_{ij}=\underset{k}{\sum }{\hat{z}}_{ik}w_{ij}^{\left[g\right]}{\hat{y}}_{kj}. \end{array}$$

The underlying assumption for Equation (6) is that the more paths connecting a lincRNA and disease, the more likely it is to be a true association. Transforming Equation (6) into matrix form, we obtain

(7) $$\begin{array}{r} X=\hat{Z}W^{\left[g\right]}\hat{Y}. \end{array}$$

Transforming Equation (7) into an optimization problem, we obtain

(8) $$\begin{array}{r} O_{G^{\left[g\right]}}=\frac{1}{2}||X-\hat{Z}W^{\left[g\right]}\hat{Y}|{|}_{F}^{2}. \end{array}$$

Because we use NMF to approximate *X*, Equation (8) is re-written as

(9) $$\begin{array}{r} O_{G^{\left[g\right]}}=\frac{1}{2}||BF-\hat{Z}W^{\left[g\right]}\hat{Y}|{|}_{F}^{2}. \end{array}$$

Combining Equations (4,5), and (9), the objective function of the proposed algorithm is defined as

(10) $$\begin{array}{l} O=O_{NMF}+\alpha O_{G^{\left[l\right]}}+\beta O_{G^{\left[g\right]}}, \end{array}$$

where parameter α, β control the contributions of two terms $O_{G^{\left[l\right]}}$ and $O_{G^{\left[g\right]}}$. The disease-lincRNA prediction problem is transformed into an optimization problem as

(11) $$\begin{array}{ll} \underset{B,F}{\min} & \frac{1}{2}||\hat{X}-BF|{|}^{2}+\alpha Tr\left(B^{\prime }L^{\left[l\right]}B\right)\\ \;+\frac{\beta }{2}||BF-\hat{Z}W^{\left[g\right]}\hat{Y}|{|}_{F}^{2}\\ s.t.\;B\geq 0,F\geq 0. \end{array}$$

In the next subsection, we address how to optimize the problem in Equation (11).

### 2.3. Optimization

An iterative two-step strategy is adopted because direct optimization to Equation (11) is difficult, where we optimize matrices *B* and *F* by fixing parameters. At each iteration, either matrix *B* or *F* is optimized first, whereas the other is fixed. Iteration is repeated until the algorithm converges or the maximum number of iterations is reached.

Let the objective function of Equation (11), i.e.,

(12) $$\begin{array}{l} L=\frac{1}{2}||\hat{X}-BF|{|}^{2}+\alpha Tr\left(B^{\prime }L^{\left[l\right]}B\right)\\ \;+\frac{\beta }{2}||BF-\hat{Z}W^{\left[g\right]}\hat{Y}|{|}_{F}^{2}. \end{array}$$

We handle the non-negative constraints for matrices *B* and *F* using the Larange method. Specifically, let ϕ_*ij*_ and ψ_*ij*_ be the Larange multiplier for the constraints *b*_*ij*_ and *f*_*ij*_, respectively. Considering Φ = [ϕ_*ij*_], Ψ = [ψ_*ij*_], the Larange L of Equation (12) can be formulated as

(13) $$\begin{array}{l} L=\frac{1}{2}||\hat{X}-BF|{|}^{2}+\alpha Tr\left(B^{\prime }L^{\left[l\right]}B\right)\\ \;+\frac{\beta }{2}||BF-\hat{Z}W^{\left[g\right]}\hat{Y}|{|}_{F}^{2}+\Phi B+\text{Ψ}F. \end{array}$$

The partial derivatives of L with respect to basis matrix *B* and feature matrix *F* are calculated as

(14) $$\begin{array}{l} \frac{\partial L}{\partial B}=\left(1+\beta \right)BFF^{\prime }-\hat{X}F^{\prime }+2\alpha L^{\left[l\right]}B-\hat{Z}W^{\left[g\right]}\hat{Y}F^{\prime }+\Phi , \end{array}$$

and

(15) $$\begin{array}{l} \frac{\partial L}{\partial F}=B^{\prime }\hat{X}-B^{\prime }BF+\beta B^{\prime }BF-B^{\prime }\hat{Z}W^{\left[g\right]}\hat{Y}+\text{Ψ}. \end{array}$$

According to the Karush-Kuhn-Tucker conditions ϕ_*ij*_*b*_*ij*_ = 0 and ψ_*ij*_*f*_*ij*_ = 0, we obtain the updated rules

(16) $$\begin{array}{l} B=\frac{\hat{X}F^{\prime }+\hat{Z}W^{\left[g\right]}\hat{Y}F^{\prime }}{\left(1+\beta \right)BFF^{\prime }+2\alpha L^{\left[l\right]}B}B, \end{array}$$

and

(17) $$\begin{array}{l} F=\frac{B^{\prime }BF+B^{\prime }\hat{Z}W^{\left[g\right]}\hat{Y}}{B^{\prime }\hat{X}+\beta B^{\prime }BF}F. \end{array}$$

The Cr-NMF algorithm is presented in Algorithm 1.

**Algorithm 1:** The Cr-NMF algorithm

**Input:**
*G*^[*l*]^: Co-expression network for lincRNAs; *M*^[*g*]^: Expression profile for genes; *M*^[*l*]^: Expression profile for lincRNAs; $\hat{X}$: Known disease-lincRNA associations; $\hat{Y}$: Known disease-gene associations; α, β: Parameters control relevant importance.
**Output:**
*X*: Predicted disease-lincRNA associations. ***Step 1: Data Processing***
1: Construct co-expression network *G*^[*l*]^ for lincRNAs using expression profile *M*^[*l*]^;
2: Construct gene-lincRNA associations $\hat{Z}$ using *M*^[*l*]^ and *M*^[*g*]^;
3: Construct Laplacian matrix *L*^[*g*]^ for *G*^[*g*]^;
4: Construct Laplacian matrix *L*^[*l*]^ for *G*^[*l*]^; ***Step 2: Matrix Factorization***
5: Make initial matrices B and F;
6: Update matrix *B* according to Equation (16);
7: Update matrix *F* according to Equation (17);
8: Goto Step 5 until the algorithm is convergent; ***Step 3: Predict disease-lincRNA associations***
9: Predict disease-lincRNA association as *X* = *BF*;
10: **return** *X*

### 2.4. Algorithm Analysis

The complexity of algorithm is investigated. On the space complexity of algorithm, the space for the gene genetic network is $O\left(n_{g}^{2}\right)$. The space for lincRNA co-expression network is $O\left(n_{l}^{2}\right)$. The space of disease-lincRNA association, disease-gene associations, and gene-lincRNA association is *O*(*n*_*d*_*n*_*l*_), *O*(*n*_*d*_*n*_*g*_), and *O*(*n*_*g*_*n*_*l*_), respectively. The space of basis matrix *B* and feature matrix *F* is *O*((*n*_*l*_ + *n*_*d*_)*r*), where *r* is the number of latent variables. Thus, the total space of Cr-NMF is $O\left(n_{l}^{2}+n_{g}^{2}+n_{d}n_{l}+n_{d}n_{g}+n_{g}n_{l}+\left(n_{l}+n_{d}\right)r\right)$. Because *n*_*d*_ ≪ *n*_*g*_ and *n*_*l*_ ≪ *n*_*g*_, the total space of the proposed method is $O\left(n_{g}^{2}\right)$.

The running time of the proposed algorithm depends on the updating rules in Equations (16) and (17). Thus, the time complexity of Cr-NMF is the same as that of NMF, i.e., *O*(*tkn*^2^), where *t* is the number of iteration (Lin, 2007). Thus, the overall running time for RNMF-MM is *O*(*tkn*^2^) + *O*(*n*^2^) = *O*(*tkn*^2^), indicating that the proposed algorithm is also efficient in comparison with the NMF algorithm.

## 3. Results

In this section, we validate the performance of the proposed algorithm. The data, parameter selection as well as the performance of algorithms are addressed in turns.

### 3.1. Data

The lincRNAs are downloaded from the Human BodyMap project, which provides a catalog of lincRNAs from RNA-seq data across 22 tissues (Cabili et al., 2011). The catalog contains transcript expression profile across the tissues using the Cufflinks (Trapnell et al., 2010).

The association dataset of lincRNAs and diseases are extracted from the LncRNADisease database (Chen et al., 2012) in January 2015. There are 1564 lincRNAs and their associations with 1641 diseases. We employ the OMIM API function call (Hamosh et al., 2005) to retrieve closely matched phenotype IDs, resulting in a set of 684 OMIM phenotypes (mainly disease) associated with lincRNAs. All the diseases without matching any valid OMIM phenotype ID are removed. Finally, we obtain the lincRNA-disease association among 562 lincRNAs and 645 OMIM diseases.

The mRNA-disease associations are downloaded from DisGeNET software (Bauer-Mehren et al., 2010), where 16,666 mRNA genes are associated with 13,135 diseases. Similar to the lincRNA-disease associations, we use the OMIM function call to map disease names to matched phenotype IDs, and only these diseases with at least one lincRNAs are selected. Finally, 180,266 gene-disease associations are obtained among 645 OMIM diseases and 13,425 coding-genes.

The gene genetic interaction network is extracted from Lin et al. (2010), where 4,836,794 interactions among coding-genes. Only these genes associated with at least one disease are retained, resulting 3,264,923 interactions among 13,425 genes.

In this study, we want to make use the connections between lincRNAs and coding-genes. Based on Biswas et al. (2015), we construct the lincRNA-gene association network from diseases. Specifically, if the *i*-th lincRNA is connected to the *j*-th coding-gene if and only if both of them are associated with at least a disease. Based on this strategy, there are 1,775,375 edges among 562 lincRNAs and 13,425 coding-genes.

### 3.2. Settings

To fully validate the performance of the proposed algorithm, we select five well-known algorithms for a comparative comparison: NMF (Lee and Seung, 1999), non-smooth NMF (nsNMF) (Pascual-Marqui et al., 2001), integrated NMF (iNMF) (Biswas et al., 2015), Label Propagation (LP) (Hwang et al., 2011), and Random Walk (RW) (Li and Patra, 2010). All these algorithms can be categorized into two classes: matrix decomposition based and topological structure based methods. The matrix decomposition-based algorithms include NMF, nsNMF, and iNMF, while the topological structure-based methods are LP and RW.

To evaluate the performance of these algorithms, three measures, including mean absolute error (MAE), Accuracy and root mean squared error (RMSE), are employed to quantify the accuracy of algorithms. They are defined as Herlocker et al. (2004):

(18) $$\begin{array}{l} MAE\left(\hat{X},X\right)=\frac{1}{\left|\tau \right|}\underset{\left(i,j\right)\in \tau }{\sum }|{\hat{x}}_{ij}-x_{ij}|, \end{array}$$

(19) $$\begin{array}{l} Accuracy\left(\hat{X},X\right)=1-MAE\left(\hat{X},X\right), \end{array}$$

(20) $$\begin{array}{l} RMSE\left(\hat{X},X\right)=\sqrt{\frac{1}{\left|\tau \right|}\underset{\left(i,j\right)\in \tau }{\sum }{\left(\left|{\hat{x}}_{ij}-x_{ij}\right|\right)}^{2}}, \end{array}$$

(21) $$\begin{array}{l} RSS\left(\hat{X},X\right)=\sqrt{\underset{i,j}{\sum }{\left(\left|{\hat{x}}_{ij}-x_{ij}\right|\right)}^{2}}, \end{array}$$

where $\hat{X}$ and *X* are the observed association matrix and the predicted association matrix, respectively. τ is the set of lincRNA-disease association for prediction, i.e., τ is considered as the test set.

### 3.3. Parameter Selection

Three parameters are involved in the proposed algorithm, where parameter α determines the relevant importance of lincRNA co-expression networks, parameter β controls the relevant importance of the gene genetic network, and parameter *k* is the number of features for the basis and coefficient matrices. Similar to Ref., we set α = β by assuming that the lincRNA co-expression network and gene genetic network are equally important in discovering the lincRNA-disease associations.

**Figure 3:**
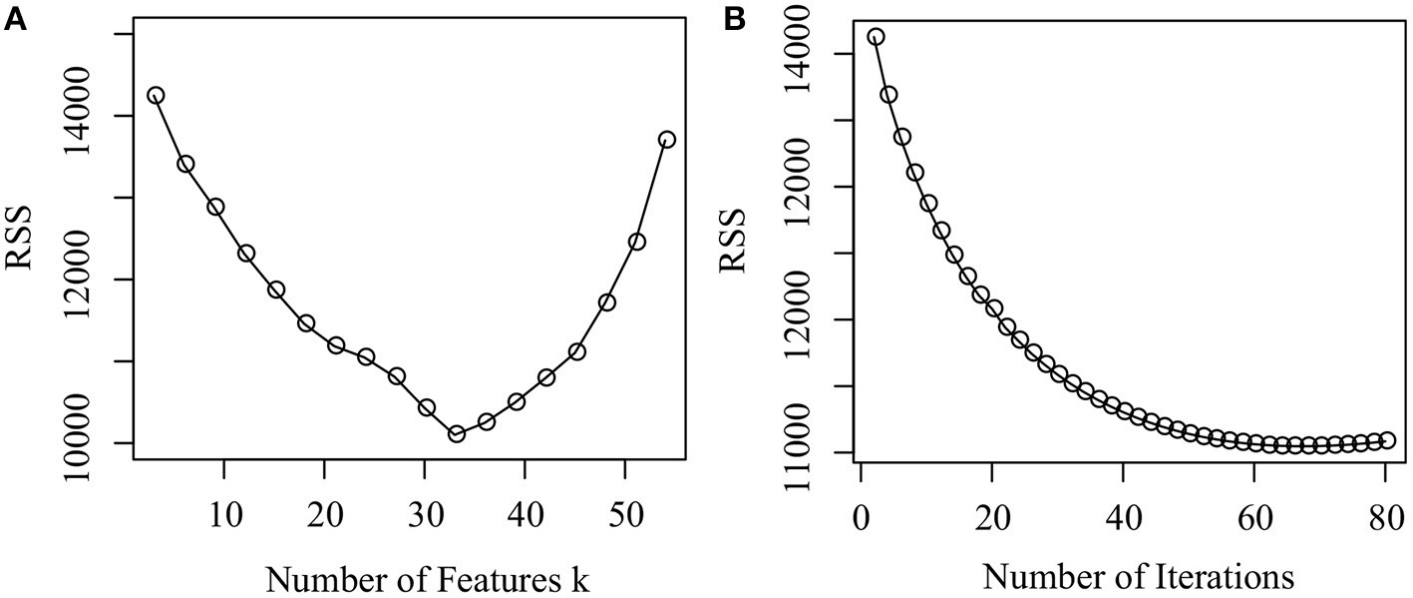
Parameter selection and convergence analysis. **(A)** How the RSS changes as the number of features changes from 3 to 54, and **(B)** How the RSS changes as the number of iterations increases from 1 to 100.

We first investigate how parameter *k* determines the performance of the proposed algorithm. Figure 3A illustrates how RSS changes from 3 to 54 with a gap 3. From Figure 3A, we conclude that as *k* increases from 3 to 33, RSS dramatically decreases, which implies that the accuracy of the proposed algorithm increases. As *k* increases from 34 to 54, RSS increases. There is a good reason why this occurs. When *k* is small, the number of the latent features is insufficient to characterize the lincRNA-disease associations. When *k* is large, the number of the latent features is redundant. *k* = 33 reaches a good balance between them since RSS reaches the minimum. In the experiment, we set *k* = 33.

**Figure 4:**
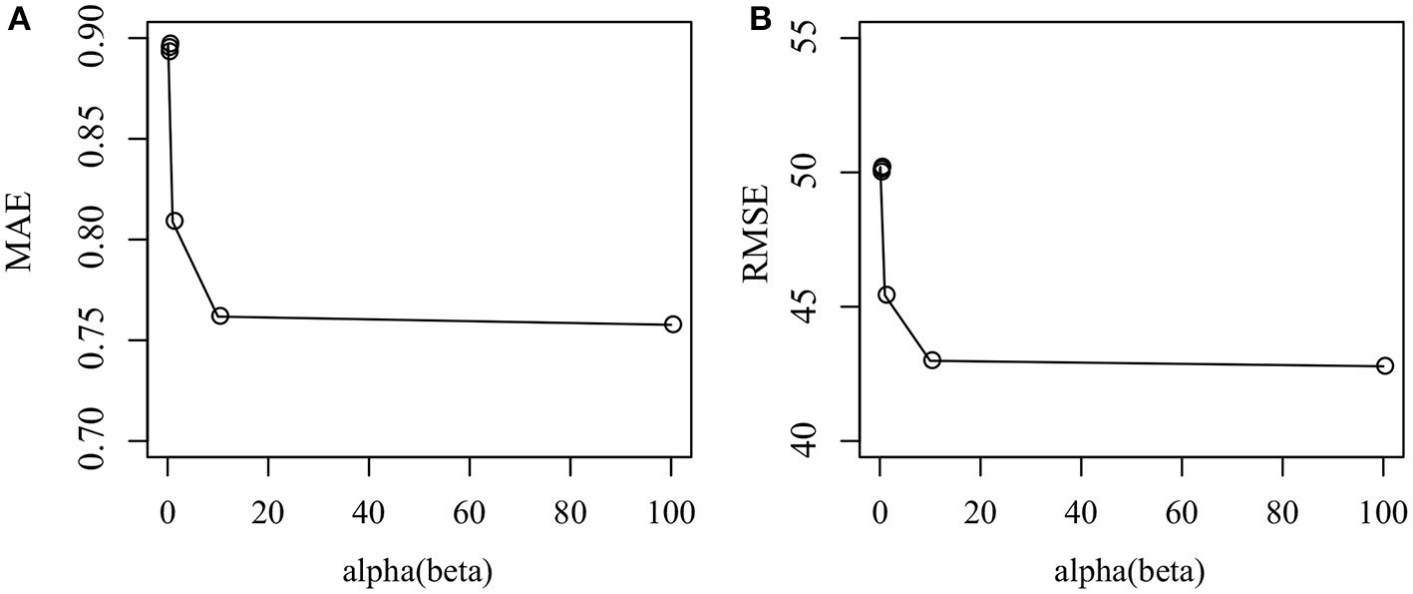
How parameter affects the performance of the proposed algorithm in terms of various measurements: **(A)** MAE, and **(B)** RSME.

We then investigate how parameter α and β affect the performance of the Cr-NMF algorithm. Figure 4 shows that how MAE and RMSE change as α ∈ {0.001, 0.01, 0.1, 1, 10, 100}. It is shown that the proposed algorithm achieves the best performance when α = 1. Furthermore, the proposed algorithm is robust since the perturbation of performance is subtle if α ∈ [10, 100], indicating that Cr-NMF is not sensitive to parameter α and β. Even though MAE and RMSE decrease when α ∈ [10, 100], the change is subtle.

Finally, we check the convergence of the proposed algorithm. Figure 3B shows how RSS changes as the number of iterations increases. It is easy to assert that, when the number of iterations reaches 60, the algorithm converges because *RSS* does not change dramatically any more. Thus, the number of iterations is set as 60 in the experiments. The result demonstrates that the proposed algorithm is efficient.

### 3.4. Performance of Various Algorithms on Predicting lincRNA-Disease Associations

By setting α(β) = 10, *k* = 33, and the number of iterations as 60, we apply Cr-NMF to the omic data to predict the lincRNA-disease associations. To quantify the performance of various algorithms, the accuracy in Equation (19) is adopted, where it is also used in Biswas et al. (2015). Because all of these compared algorithms have a factor of randomness, we get rid of randomness of algorithms by running each algorithm 50 times, and the mean of accuracy is used to quantify the performance of algorithms.

**Figure 5:**
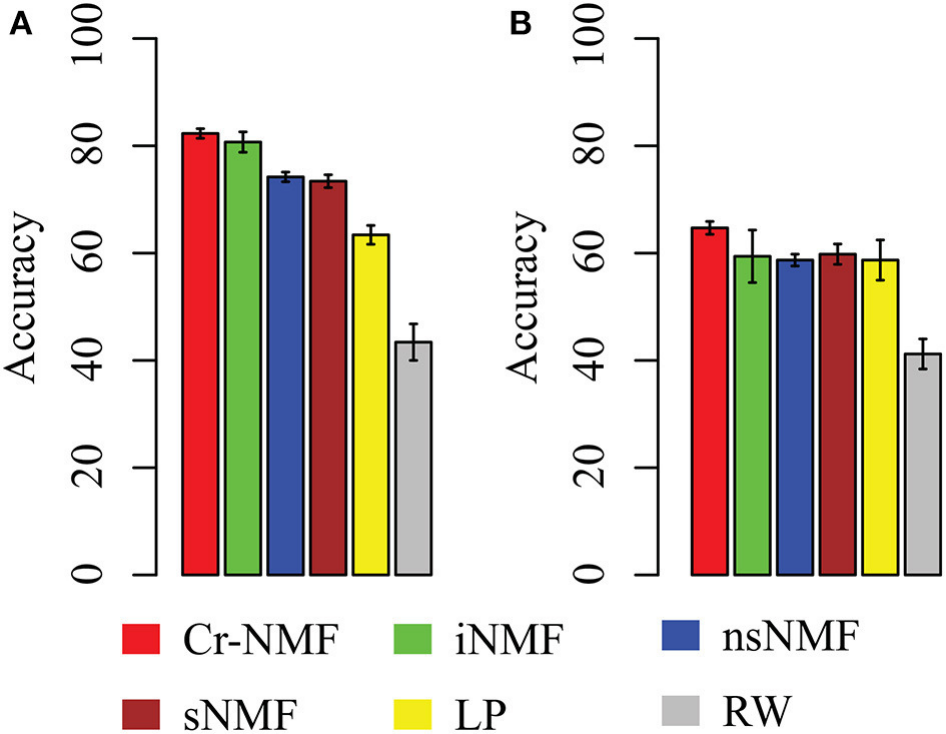
The accuracy of various algorithms on predicting disease-lincRNA associations in terms of various strategies: **(A)** leave-one-out cross validation (LOOCV), and **(B)** external validation, where Y-axis denotes mean accuracy and error bar represents standard deviation.

The leave-one-out cross validation (LOOCV) is adopted to measure the accuracy of each algorithm. Specifically, for each disease, we remove all the associations between the disease and lincRNA genes. The accuracy of various algorithms is depicted in Figure 5A. It is easy to draw conclusions such as: (1) the Cr-NMF algorithm achieves the best performance in LOOCV, followed by the iNMF algorithm. In detail, the accuracy of Cr-NMF is 0.823 ± 0.009, which is 1.9% higher than the iNMF algorithm on predicting disease-lincRNA associations. (2) Both Cr-NMF and iNMF algorithms outperform the rest of the methods, implying the integration of omic data is promising on predicting disease-lincRNA associations. Moreover, (3) The random walk and label propagation algorithms are worst in terms of accuracy. There are two reasons why the proposed algorithm outperforms the other approaches. First, the Cr-NMF algorithm directly factorize associations between diseases and lincRNAs, which captures the latent features to characterize the disease-lincRNA associations. Second, the factorization preserves the paths from “disease ⇀ lincRNA → protein-coding gene,” which more precisely infers disease-lincRNA associations. The RW and LP algorithms are much worse than the others, implying that the topological structure is insufficient to characterize the relations between diseases and lincRNAs.

In order to further validate the performance of the proposed algorithm, we take the disease-lincRNA associations before 2015 January as training set, and set the data between 2015 and 2017 July as testing set, as shown in Figure 5B. It is easy to assert that the proposed algorithm is best, followed by iNMF. Specifically, the accuracy of algorithms is 0.647 (Cr-NMF), 0.594 (iNMF), 0.587 (nsNMF), 0.598 (sNMF), 0.575 (LP), 0.412 (RW). Careful comparison between Figures 5A,B indicates that the accuracy of various algorithms in the external validation decreases dramatically. However, the relative performance of these algorithms is similar. The results demonstrate that the proposed algorithm is promising in predicting disease-lincRNA associations.

## 4. Conclusion

LncRNAs are critical regulators in human diseases and disorder pathways. Thus, it is necessary to understand the associations between lncRNAs and diseases since these relations shed light on revealing the mechanisms of complex diseases. Compared to the protein-coding genes, a very little is known about the associations of lncRNAs and diseases. The next generation of sequencing technique discovers novel lncRNAs at an unprecedent speed. Therefore, there is a critical need to develop sophisticated computational tools to predict the relations between lncRNAs and diseases.

In this study, we proposed an NMF-based algorithm to predict lincRNA-disease associations by integrating multiple types of interaction data, such as co-expression interactions between lincRNAs, disease-lincRNA associations, disease-gene associations, gene genetic interactions, and lincRNA-gene links. There are two advantages of the proposed algorithm. First, it is able to explain each of the associated lincRNA as well as disease in a latent feature space. Second, the proposed algorithm takes the path from lincRNA to disease, i.e., “disease ⇀ lincRNA → protein-coding gene,” which improves the accuracy of the prediction. The results demonstrate that the propose method outperforms state-of-the-art algorithms in terms of accuracy.

There are some limits in the proposed algorithm. First, there are two parameters involved in the methods and we solve this issue by a step search strategy in the experiments. A better and faster way to accomplish this needs to be developed. Particularly, how to infer the values of parameters by making use of the biological knowledge in diseases is ideal. Second, even though the proposed algorithm integrates omics data, incorporating additional data, such as disease networks, mutation data in genes would obtain even more meaningful results. In a future study, we will address these issues.

## Data Availability Statement

Publicly available datasets were analyzed in this study. This data can be found at: TCGA.

## Author Contributions

YL and XM constructed the original idea and designed the experiments. XM wrote the manuscript. YL proofread the manuscript. All authors contributed to the article and approved the submitted version.

## Conflict of Interest

The authors declare that the research was conducted in the absence of any commercial or financial relationships that could be construed as a potential conflict of interest.
